# Pilar cyst on the dorsum of hand: A case report and review of literature

**DOI:** 10.1097/MD.0000000000021519

**Published:** 2020-07-31

**Authors:** Meng Liu, Haicheng Han, Yan Zheng, Shengxiang Xiao, Yiguo Feng

**Affiliations:** a Department of Dermatology, the Second Affiliated Hospital of Xi’an Jiaotong University, Xi’an; b Haicheng Hospital, Weinan, China.

**Keywords:** histological features, opisthenar, pilar cyst

## Abstract

**Rationale:**

Pilar cyst mainly occurs on the scalp, but pilar cyst on the dorsum of hand has not been reported. Herein, we provide information to improve the clinical cognition of pilar cyst location.

**Patients concerns:**

A 76-year-old man presented with a round nodule on the opisthenar of his right hand for two months without any subjective symptoms.

**Diagnoses:**

Histological features of the lesion biopsy indicated the diagnosis of pilar cyst.

**Interventions:**

Surgical resection was made under local anesthesia.

**Outcomes:**

Complete recovery was achieved after surgery.

**Conclusion:**

Pilar cyst rarely occurs on the dorsum of hand and its diagnosis depends on histopathological examinations. Surgical resection is the only way to treat it.

## 1 Introduction

Pilar cysts are identified by Pinkus as the keratinization of the outer root sheath of hairs,^[1]^ which were originally called sebaceous cysts. The cysts are characterized by smooth, round nodules with solid texture and good mobility. It cannot be distinguished from epidermal cysts clinically, except that 90% of pilar cysts are found on the scalp where hair follicles are abundant. Other less common locations include face, trunk and extremities. Lesions rarely arise in palms, genitalia, axillary and groin.^[2]^ As far as we know, pilar cyst on the hand has rarely been reported before. The present study reports one such case on dorsum of hand in a male patient.

## 2 Case report

**Figure 1:**
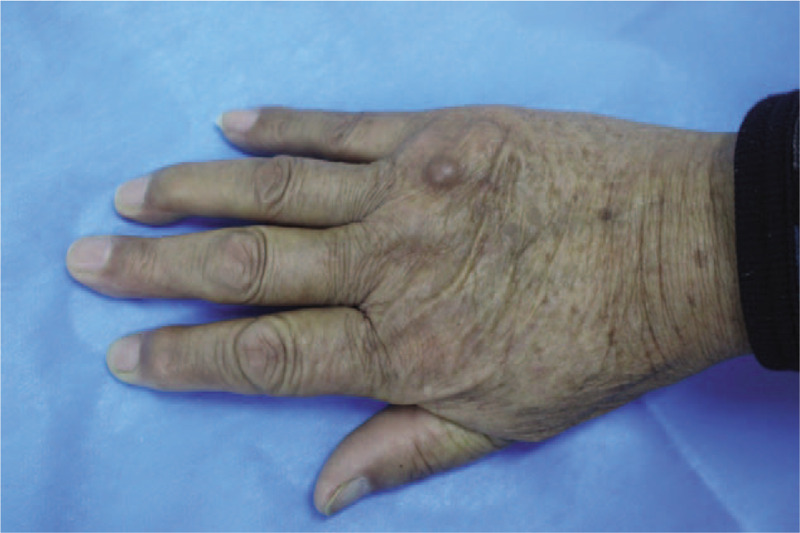
Appearance of the lesion on the back of right hand.

A 76-year-old man presented with a flesh-colored, dome-shaped nodule on the opisthenar of his right hand (Fig. 1), which had gradually increased in size for two months. The patient did not have any subjective symptoms. There is no history of trauma or chronic irritation at the site of the lesion and no similar lesion in his family members. He did not receive any treatment before he came to our hospital. The patient is generally in good condition. No changes in diet, sleep, urination, defecation, or body weight could be found. Physical examination showed that the patient possessed stable vital signs. The systematic examination revealed no evident abnormalities. A dermatological examination revealed a skin-colored nodule with smooth surface on dorsum of his right hand. This soybean-sized nodule was tough in texture with clear boundary and no hair follicle.

### 2.1 Auxiliary examination

**Figure 2:**
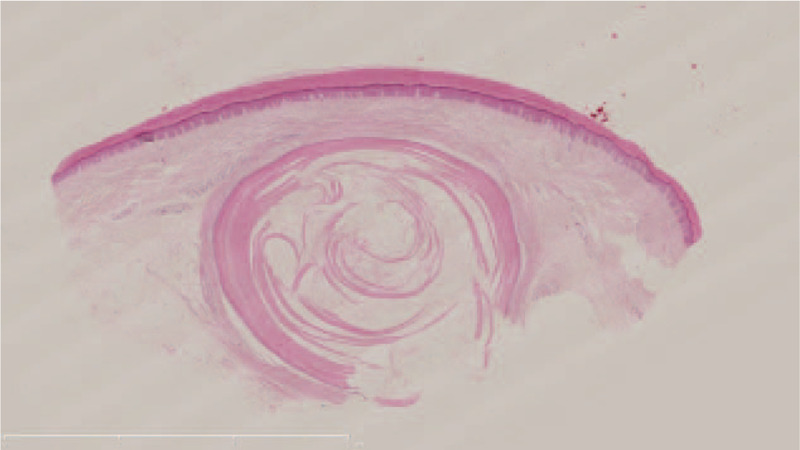
Hematoxylin-eosin (H&E) staining of the lesion, showing a cyst in the reticular dermis and eosinophilic material. Bar length = 6 mm.

**Figure 3:**
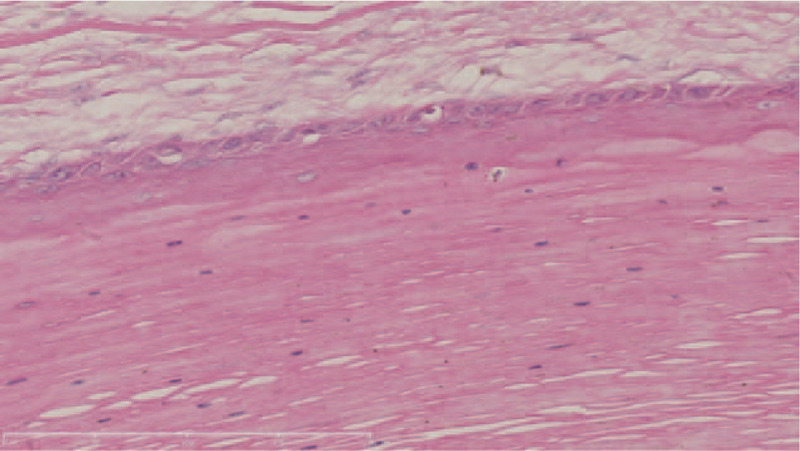
No granular layer is observed at the wall of cyst (H&E). Bar length = 200 μm.

There were no obvious abnormalities in blood routine, coagulation and liver and kidney function. A histopathological examination was conducted and the pathology showed that the cyst was in the reticular dermis (Fig. 2), with surrounding basal cells arranged as a fence. The cytoplasm of cells above the basal layer was lightly stained, swollen, and eosinophilic with fuzzy borders. The cells of stratified epithelium lost their nuclei abruptly without an intermediated granular cell layer. The innermost cells seemed to have fallen off into the cavity (Fig. 3).

### 2.2 Diagnosis

Clinically, it was considered as dermatofibroma at first. Based on histopathological examination, a diagnosis of pilar cyst was made.

### 2.3 Therapeutic intervention and follow up

Complete excision under local anesthesia was done. After the treatment, the patient achieved complete recovery with no relapse during the 6 months of clinical follow-up.

## 3 Discussion

Pilar cyst is derived from external root sheath of the follicular isthmus, which is also referred to as isthmus-catagen cyst. Pilar cyst accounts for 20% of epithelial cysts and the others are epidermal.^[3]^ Compared with epidermal cysts, the lesions of pilar cyst do not have an overlying punctum and tend to be more mobile and firmed. Clinically, pilar cyst is indistinguishable from epidermal cyst, histopathological examination is indispensable to make a definite diagnosis. Pilar cysts are intradermal cysts with distinctive histological features. The cells of the cyst wall are composed of epithelial cells without obvious intercellular bridges. The inner cells suddenly transform into solid eosinophilic-staining keratin with no intervening granular layer. Nuclei of cells that fall into the cystic cavity generally disappear, but some cells still have nuclei. The cyst contents are homogeneous. Calcification exists in about 25% of cases. If the capsule wall is ruptured, a granulomatous reaction may occur. The cyst can therefore partially or completely disintegrate.^[4]^

Pilar cyst often occurs in middle age with an obvious female preference and inheritance pattern of multiple cysts manifests as autosomal dominance.^[5,6]^ Shimomura et al et al propose a monoallelic mutation in phospholipase C delta 1 (PLCD1) that result in formation of multiple pilar cysts.^[7]^ Although pilar cyst is derived from follicular, several cases have indicated that pilar cyst can arise in non-hair bearing areas. The onset of pilar cyst was thought to be induced by inflammation and trauma.^[8–10]^ In addition, infection of the human papilloma virus may be related to the pathogenesis of pilar cyst.^[9]^ In very rare cases, pilar cyst can develop into malignant proliferative lump which clinically present as progressively enlarged lobulated masses, similar to squamous cell carcinoma.^[11]^ It is very important to distinguish pilar cysts from proliferating pilar cysts,^[12]^ since the later can undergo malignant transformation. Complete excision of the cyst is curative, but it is not recommended to remove surgically when the cyst is inflamed. Proliferating pilar cysts might need radiation therapy and/ or chemotherapy after surgical excision^[4]^.

The specificity of our case lies in its location. Compared to the incidence of scalp, pilar cyst on hand is extremely uncommon. To our knowledge, there are four cases of pilar cyst reported on hand previously, of which three are in finger tips and the other one is on the dorsum of the thumb. They were all proximal to the nail bed, which aroused the speculation that pilar cyst may be derived from nail matrix.^[13–16]^ The pilar cyst of our patient arose in the dorsum of right hand, a rare location and away from nail bed. No hair follicle was found. In other words, it can neither originate from hair follicles nor from nail matrix. Our case urges rethinking of the origin of pilar cyst. Meanwhile, it reminds dermatologist that a similar lesion on the dorsum of hand can not rule out the possibility of pilar cyst.

## Author contributions

ML and YF diagnosed and treated the patient. HH, YZ and SX conducted histopathological examination. ML and YF wrote the manuscript. All authors have approved the final article be true.
